# From a Measure of Confidence to a Measure of the Level of Knowledge

**DOI:** 10.5334/pb.1332

**Published:** 2025-05-22

**Authors:** Daniel Defays

**Affiliations:** 1 Faculty of Psychology, Logopedics and Educational Sciences, University of Liège, B4000 Liège, Belgium

**Keywords:** confidence, calibration, cognitive bias, error estimation, meta cognition

## Abstract

Confidence degrees assigned by respondents to their responses are generally taken at their face value. An experiment where respondents were asked to indicate twice their confidence in their (changed or unchanged) response has, however, showed that those confidences can greatly vary over time at the individual level. I propose a model that takes that variation into account and considers confidence as a latent variable – the level of knowledge – to be estimated through a true score approach. The model is defined in the special case of a scale with a given number of confidence degrees. It assumes that when faced with this type of testing requirements, a person experiences uncertainty in a way that can be represented by a finite set of partial knowledge states. It leans mainly on a conditional independence assumption. As the model is intractable under that sole assumption, additional testable and simple constraints must be imposed on the way confidence errors are distributed. The model was applied to data collected in the experiment. The results show that, under a general (population) overestimation bias, very different individual profiles are hidden with different distributions of errors. The model enables also to make predictions about one single individual by only examining his (her) calibration errors. Some errors patterns observed on the replicated data can indeed be anticipated with the proposed models.

The ability to judge one’s performance has been shown to play a key role in various fields such as medical diagnoses, financial investing, eyewitness identification, psychological testing, and education (Leclercq, 1982; Merkle & Van Zandt, 2006; Koriat & Adiv, 2015). In education and psychological research, this ability typically manifests as an assessment of one’s confidence in having provided the correct answer. Various indicators have been developed to measure respondents’ ability to realistically estimate the probability of their response being correct and to identify different types of biases. In these approaches, confidence levels are generally accepted at face value, without considering their intra-personal variability. However, several experiments have shown that when respondents are asked to indicate their confidence in their response twice, these confidence levels can vary (Lichtenstein, Fisschoff & Phillips, 1952; Budescu, Wallsten & Au, 1997; Koriat, 2011; Koriat & Adiv, 2015; Leclercq, 2016). For instance, an experiment conducted by Leclercq (2022) demonstrated that in more than 50% of cases, on a scale with six levels of confidence, respondents changed their confidence level.

In this paper, I propose a model that accounts for this variation and considers confidence as a latent variable to be estimated through a true score approach. The model focuses on scales with a limited number of degrees (e.g., six) and considers confidences (judgments of correctness) expressed in percentages. This is merely an analysis constraint; continuous judgments can be collected and subsequently rounded or binned for analysis. The model explicitly addresses the error made by a subject in estimating their “true” level of knowledge (LOK, the latent variable). It assumes that under these testing conditions, the uncertainty experienced by the subject can be represented by a finite set of partial knowledge states. Given a simple assumption of conditional independence—the probability of giving a correct answer depends on the subject’s LOK but not on the error made in estimating that LOK—the model, with some testable and simple additional assumptions on the distribution of errors, leads to the resolution of a system of nonlinear equations under constraints. This approach enables the maximum likelihood estimation of key parameters, such as the probability of overestimating or underestimating confidence for different LOKs and the distribution of LOKs.

This approach offers several benefits. Firstly, it primarily (but not exclusively) assumes conditional independence, whereas other models typically involve more assumptions. Secondly, it allows for the identification of traits at the individual level, with each person being modelled separately. Thirdly, it provides a detailed view of miscalibration. Well-known effects such as over/underestimation and hard/easy effects are shown by this model to be features of more general misclassification patterns, potentially leading to better strategies for correction where needed. Additionally, studies on the stability of biases over time can benefit from a more comprehensive view of biases. The model enables detailed investigations into the links between biases and fields of knowledge, individual traits, or demographic variables, thanks to accurate estimates of different types of biases. Fourthly, the use of a true score approach allows for the linking of errors over time, observed when measurements are repeated, to confidence accuracy. This facilitates new predictions about how an individual will behave if faced with the same situation again. The use of replicated data makes it possible to detect interesting individual traits of confidence judgments that may not be observable with a single test. For instance, some respondents may exhibit a tendency to repeat extreme confidence levels (e.g., 50% or 100% in two-alternative forced choice (2AFC) questions) more often than other values. Others may show an attraction to the 100% confidence level, jumping from an initial confidence degree (e.g., 70%) to 100% more frequently than to any other level. In the approach adopted in this paper, these effects, typically observable only through test repetition, can be predicted from the analysis of data from a single test. Finally, the proposed model also enables the estimation of what can be termed the “knowledge landscape” (or profile) of each respondent, without requiring additional estimates of item difficulty (as in Item Response Theory; IRT). The knowledge landscape of a respondent represents the distribution of items in a given domain according to their levels of knowledge.

## Paper organization

Before presenting the true score approach in detail, I will briefly review existing models of the cognitive process leading to a judgment of confidence and the measurement of inaccuracies in confidence judgments. A theory of error requires a sound foundation of the notion of confidence. For instance, the confidence expressed in statements like “I think Mozart died in the second half of the seventeenth century with a confidence degree of 80%” needs to be clearly modeled. Here, I will propose—or recall, as there is nothing novel in my approach—a simple framework to measure confidence without assuming a specific underlying cognitive process. A classical model of the measurement error of LOK assessments is then developed for the special case of a scale with a given number of confidence degrees. Through assumptions of conditional independence and of different types of errors, a method to estimate LOK and the probability of errors is derived. Statistical tests are proposed to compare different models.

The data of an experiment in which students are asked twice to provide their confidence levels in their responses to a test of 60 two-alternative forced choice (2AFC) questions will be analyzed using true score models to illustrate their potential benefits. The different models are designed to validate well-known (but sometimes questioned) empirical regularities such as overconfidence, the hard/easy effect, and the tendency to use extreme values on the confidence scale. A discussion and conclusions are presented in the final two sections.

## Measure of confidence

It is natural to compare the confidence expressed by a respondent in having provided the correct answer with the actual success rate of the corresponding responses. For instance, what is the proportion of correct responses among those given with a confidence level of 70%? Confidence accuracy, often termed calibration, has been studied for years from various perspectives. The definition of confidence degrees and scales, for example, has been investigated by Leclercq (2016, 2017). The accuracy of individuals in estimating the probability of their responses being correct is a widely covered topic in the metacognition literature. Various types of biases, such as the hard/easy effect (Lichtenstein, Fisschoff & Phillips, 1982), overconfidence bias (Fajfar & Gurman, 2009; Miller et al., 2015), and consensuality and consistency effects (Koriat & Adiv, 2015), have been detected and analyzed. Additionally, different calibration measures have been proposed (Fajfar & Gurman, 2009; Schraw et al. 2013; Fleming & Lau, 2014; Rutherford, 2017; Attali et al., 2020). However, studies on the inaccuracy of confidence judgments through the analysis of repetition errors are much less frequent.

## Modeling the cognitive process

Various mathematical models of confidence calibration have been proposed, generally based on modeling the cognitive process underlying confidence and the decision process by which a subject selects one of several alternatives. Judgments are seen as inferential in nature, formulated as a confidence level reached through a cognitive process primarily viewed as an elicitation process where evidence in favor of one of the proposed responses is accumulated. Cues, beliefs, heuristics, information from long-term memory, or elements derived from the task itself come to the respondent’s mind and favor one of the possible responses. The underlying cognitive processes may depend on the type of tasks (e.g., general knowledge tasks versus perceptual tasks) (Koriat, 2011).

To quantify this process, Gigerenzer, Hoffrage, and Kleinbölting (1991) proposed a probabilistic mental model (PMM) in which respondents test several cues until they find one that discriminates between the proposed choices. The validity of the cue (formalized as a conditional probability) is then reported as the confidence in the choice. In 1994, Erev, Wallsten, and Budescu proposed a true score approach in the form of a general stochastic model incorporating random error in probability judgments. Their model assumes that individuals experience a degree of confidence in their responses represented by a continuous variable. They use a log-odds transformation of that variable, to which an error is added, and a response rule to derive the subjective judgments expressed by individuals. In this model, the errors are independent of the experienced degree of confidence and are normally distributed. It also applies when response categories are imposed. Merkle and Van Zandt (2006) assumed a Poisson Race model to represent the cognitive process. In their model, the subject accumulates information favoring the two possible responses on two separate counters. Once a threshold is reached in one of the counters, the corresponding response is given.

All these models represent confidence as a random variable whose value depends on the particular measurement situation. When the measurement is repeated, other values can be observed. To my knowledge, most models are used to explain the discrepancies between confidence judgments and what Erev et al. (1994) called true judgments. Few attempts have been made to compare these discrepancies with repetition error. Some authors, such as Budescu, Wallsten, and Au (1997), have highlighted the importance of within-person/item variability. However, their model requires repeated measurements to disentangle repetition error from other sources of variation. Additionally, they rely on sophisticated response rules, strong distributional assumptions (e.g., random distribution of errors), and postulate the independence of the error from the item.

## Biases in confidence assessments

Other models, based on similar elicitation processes, have been proposed to capture biases observed in confidence assessments. Indeed, several biases in the metacognitive process leading to the expression of a confidence level have been identified. Numerous experiments have shown that, in certain domains (e.g., tests of attitude, general knowledge, perception, personal preferences), responses that are highly consensual (even if incorrect) are associated with high confidence; this is known as the consensuality principle. Its within-subject counterpart is the consistency effect: when asked the same question multiple times, subjects tend to maintain consistency in their responses and assign higher confidence to the most frequent answer (Koriat, 2011; Koriat & Adiv, 2015), particularly in general knowledge tasks, perceptual judgments, and social attitudes tests. When subjective performance expectations exceed (or fall short of) actual performance, individuals are classified as overconfident (or underconfident) (Fajfar & Gurman, 2009). The hard/easy effect refers to the tendency to overestimate one’s chances of success in difficult tasks (Lichtenstein et al., 1982) and to underestimate them in easy tasks.

Theories and models that have been proposed address these effects. Underconfidence is explained as an attempt to avoid the overconfidence knowledge state, perceived as a “loser” state (Fajfar & Gurman, 2009). Other authors suggest that the hard/easy effect and overconfidence could be linked to researchers’ failures to sample items representative of the natural environment (see Koriat, 2018, for a review). For instance, Gigerenzer (2004) conjectures that confidence in one’s knowledge is largely determined by the structure of the task and the corresponding known environment in a person’s long-term memory. Questions that are too difficult (not representative of the known environment) will lead to overestimation of confidence. Winman, Juslin, and Björkman (1998) provided another explanation. Overconfidence could result from a process occurring after the elicitation process: a kind of a posteriori accuracy assessment of the probability of being correct. Individuals ask themselves, “Given my state of information, would I have been able to select the correct answer?” A hindsight bias in past experience would then cause overconfidence. The consensuality effect arises from the fact that people largely draw representations from the same population of representations associated with an item, while the consistency effect stems from increasing subjective confidence in responses eliciting the same type of cues, in other words from a focus on reliability more than on validity. Koriat and Adiv (2015) proposed a quantitative model to explain these biases. In their model of subjective confidence, when faced with a question, participants sample a few representations from a population of potential representations associated with the item. Once a choice is made, confidence is based on self-consistency, reflecting the general agreement among the sampled representations favoring the decision reached. It reflects the number of pros and cons associated with the choice (irrespective of their meaning and importance). Confidence is more an assessment of reproducibility (a new sample of cues would lead to the same conclusions) than the validity of a response. Tullis and Goldstone (2020), in a completely different setting—the study of the benefits of peer instruction—and from a different viewpoint, reached a related conclusion regarding the link between correctness and confidence. They observed that following peer discussion on the relevance of different possible responses to a question, the confidence of the peers increased from pre- to post-discussion more for correct answers than incorrect ones when they agreed. According to them, this indicates that the correctness of an answer carries weight beyond confidence. The link between correctness and confidence is evidently complex.

In conclusion, various theories have been proposed to explain the ability to recognize one’s own successful cognitive processing, the experimental results, and the biases observed in the accuracy of that recognition. These theories include strategies to maximize a utility function, estimation of the difficulty of an item, estimation of the validity of a cue that made a choice possible, accumulation of evidence favoring a response, sensitivity to the consistency of cues elicited by an item, and mental review of similar past experiences from which an assessment of correctness is derived. The focus has primarily been on the impact of the nature of the task on the confidence-making process rather than on specific biases linked to individuals. Consequently, we are still far from a unified theory that can assess biases and address the inaccuracies in judgments made by subjects regarding their performances. Additionally, the anticipation of what could happen if a test is repeated does not seem to be a core concern of these models.

## Measurement of subjective confidence

Indicators have been proposed to measure different features of subjective confidence, including:

- Overall biases: Estimated as the difference between accuracy (proportion of correct responses) and mean confidence.
- Sensitivity (also called resolution): The ability to distinguish between correct and incorrect judgments, quantified as the difference between the mean confidence given to correct responses and the mean confidence given to incorrect ones (Leclercq, 1982; Fleming & Lau, 2014).
- Realism (also called appropriateness or calibration index): The ability to indicate one’s confidence appropriately, calculated as the mean errors in absolute terms (or as a weighted sum of squares) between the used confidence degrees and the corresponding proportions of correct answers (Brier, 1950; Leclercq, 1982; Budescu & Johnson, 2011; Prosperi, 2015).

While these measures are simple and straightforward, more sophisticated ones have also been proposed. For instance, the Goodman-Kruskal (G) correlation coefficient between confidence and accuracy is now widely used to measure calibration, despite being shown to be contaminated by metacognitive biases (Masson & Rotello, 2009). Sensitivity indices inspired by the signal detection theory, based on a ROC (receiver operating characteristic) analysis (Fleming & Lau, 2014), have been introduced to measure sensitivity independently of possible biases. All these indicators rely on the joint distribution of accuracy and confidence. Unfortunately, the estimation of this distribution is not error-free. There is no distinction made between the ability to assess the LOK (approximated by the given confidence) and the errors made in its assessment. These errors can arise from various sources, such as the non-representativeness of the questions, the circumstances of the testing, the payoff matrix, and the tendency to guess.

Other measurement frameworks, such as IRT and logistic regression, have also been proposed to address various biases. Budescu and Johnson (2011) employed a mixed logistic regression model to incorporate different explanatory variables in the study of calibration. In 2020, Attali, Budescu, and Arieli-Attali used logistic regression to examine the dependencies between response accuracy and various dependent variables, such as subjective confidence (conceived as the subjective difficulty of an item i for a person j), a measure of the objective difficulty of the item i for the person j (estimated from a different dataset), an intercept, and two error terms—one linked to the person and the other to the item. This approach relies on several assumptions (additive effects of the dependent variables, normal distribution of errors) and cannot assess individual profiles, as it only captures “population” effects like overall bias in subjective or objective difficulty. More importantly, the model does not address the intrinsic variability of subjective confidence. Ferrando, Anguiano-Carrasco, and Demestre (2013) also used an IRT approach combined with structural equation modeling to capture the links between subjective confidence and accuracy. Similar to Attali’s approach, their model requires preliminary estimates of item difficulty (using a two-parameter logistic model), assumes linearity and additivity of effects, and estimates only a general tendency for each person to respond with a greater or lesser degree of certainty compared to others. This family of models (including factor analysis and structural equations) also requires extensive data and, unlike my approach, cannot infer anything from observations on a single person. They lead to simple characterizations of individual profiles captured by one or two parameters.

To the best of my knowledge, no attempts have been made thus far to model LOK as a latent trait measured with error—an error that can simultaneously explain miscalibration and repetition discrepancies. The study of calibration has generally been insensitive to intertrial variations. In educational settings, teachers continue to grapple with confidence levels, often taking them at face value, without any appropriate psychometric models to address their lack of accuracy.

## Confidence: Definitions and Notations

Before delving into the formalization of the problem, it is essential to establish the context. Consider a scenario where an individual, denoted as S, is presented with an open-ended question on a specific topic. For instance, the question might be: “In which century did Amadeus Mozart die?” In addition to providing an answer, the individual is required to indicate their degree of confidence in the accuracy of the response. The confidence levels are selected from a predefined set of options: 0%, 20%, 40%, 60%, 80%, and 100%.

In the following, G will denote a set of questions and S the person. I will assume that the answers given to the questions g of G are either correct or false and I will note *R_S_ (g) = 1* or *0* accordingly. *R_S_* is therefore a function of G into {0,1} that quantifies the correctness of the response given by S (sometimes just called accuracy). When there is no ambiguity, I will drop the subscript and will note *R(g)* this function.

### Definitions of confidence

Let us associate to each g a number *c_S_(g)*, which denotes the confidence S has in the correctness of the response given to g. I will assume that *c_S_(g)* belongs to C ⊂ [0,1]. *c_S_(g)* can be conceived, under an assumption given below, as a judgment of the LOK – *K_S_(g)* – by subject S of the topic covered by the question g. I will drop again the subscript S when there is no ambiguity. Note that for these measures, the measuring instrument is the individual themselves. *c(g)* and *K(g)* are functions of G into C. However, the function *K(g)* is not arbitrary. To qualify as LOK, it must satisfy the following equality:


\[
\pi \left\{{\rm g:}\ R(g)=1|K(g)\ =\ {\rm p}\right\}\ =\ {\rm p}\ {\rm for}\ {\rm all}\ {\rm p}\ \in\ {\rm C}\ {\rm such}\ {\rm that}\ \left\{g{\rm :}\ K(g)=p\right\}\ne \phi
\]

In this formula, π designates the proportion, the bar | indicates that the proportion is calculated among the questions with *K(g)* = p, and f designates the empty set. This property of *K(g)* is the classical assumption made on confidence degrees and can be formulated as follows: the proportion of correct responses (without random guessing) among questions of level of knowledge *K(g)* is *K(g)*. This means that when a person is in a state of knowledge *K(g)* (with *K(g) = p*), the probability of responding correctly to g is p. *K(g)* would be the response given by a calibrated subject. The function *K(g)* thus characterizes a state of partial knowledge—a feeling of knowing. This state of knowledge is evaluated by the proportion of questions that it allows to succeed. The purpose of this paper is to study the measure of this state of knowledge. As previously mentioned (and this is key to my approach), numerous experiences have clearly shown that individuals (a) are hardly able to perfectly evaluate their LOK without calibration error, and (b) can change the confidence given to an answer when a test is repeated under the same conditions. The measurement of the state of knowledge is therefore biased by errors that must be assessed.

**Figure 1:**
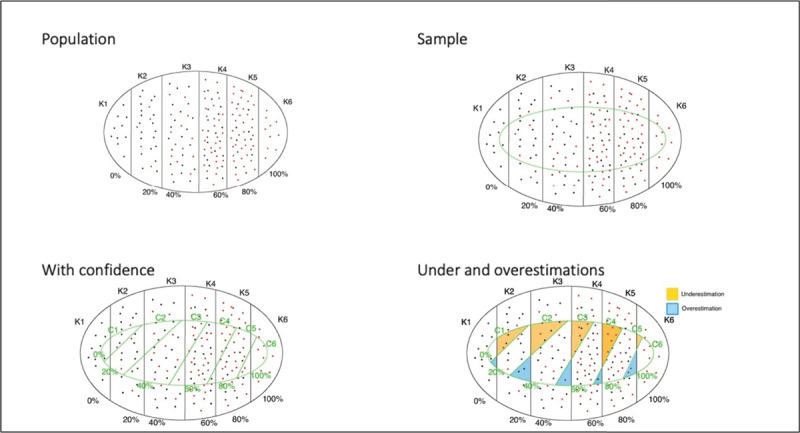
Representation of Under and Overestimations. *Note*. Top left panel: all the items of a domain known correctly (red dots) or incorrectly (black dots) by a person, distributed according to their level of knowledge. Top right panel: all the items sampled (green subset) through a questionnaire. Bottom left panel: the sample of items with their given confidence and their level of knowledge. Bottom right panel: the sample of items with, in yellow, those that are underestimated (confidence lower than level of knowledge) and, in blue, those that are overestimated (confidence higher the level of knowledge).

Figure 1, with four panels, illustrates the model. The top left panel (Population) represents the initial situation for a given domain and individual. The different elements of knowledge with their corresponding LOKs are represented by dots. These dots are red if the knowledge is correct and black if it is not. By definition of LOK, the proportion of red dots for a given level of knowledge is equal to that level of knowledge (e.g., 40% of red dots for K3). Through a questionnaire, only a sample of the population of knowledge elements is observed, as represented in the top right panel (Sample). As the different items are also characterized by a confidence level (which does not perfectly match the LOK), we encounter the situation depicted in the bottom left panel (With confidence). Given that confidences and LOKs do not coincide, we have items where confidence is lower than LOK (underestimated) and items where LOK is overestimated. This is represented in the bottom right panel (Under and overestimations).

There is another, but equivalent, way to formalize confidences. Given a set C = {c_1_, c_2_, …c_n_} of proportions ranging from 0 to 1, we assume that the set G of questions can be partitioned into n classes. Each class i of the partition is characterized by the proportion c_i_ of items to which the subject could successfully respond. In other words, the subject is assumed to have some feeling of correctness—they experience a degree of confidence in the truth of what they know (Erev, 1994). This feeling, under the given testing conditions, takes on only a limited number of values, and questions can be categorized according to the feeling they evoke. When asked to indicate their level of confidence in a response, the subject is thus able to identify (with errors that I will model) the class from which the item originates, labeled as c_i_.

### A true score model

Each individual is invited to associate a confidence degree, chosen from a set C of possible values, with the answers they provide. If I note *c(g)* the confidence degree given by the subject and *E(g)* the error in the appreciation given of their confidence, the following equality holds:


\[
c(g) = K(g)+E(g)
\]

Even when an individual provides the same answer to the same question multiple times, the confidence degree c(g) associated with their answer can vary. It is therefore more accurate to rewrite the previous equality as follows:


\[
c\left(g,\ \omega \right)\ =\ K(g)+E\left(g,\ \omega \right)
\]

with ω representing the particular measurement situation. When an individual gives their confidence degree, *c(g)* and *E(g)* can therefore be considered as random variables (question g being fixed). Estimating K(g) from a realization of c(g) is only possible if we know the distribution of the error E(g). This is the problem I now propose to tackle.

## Estimation of the LOK

### Derivation of the two basic formulas

Let us suppose that we randomly draw a question g from G. The individual to whom the question is asked is invited to complement their answer with a confidence degree chosen from a limited set of possibilities. In this situation, two sources of randomness are combined: the sampling of a question g from G and the choice of a particular situation from all the situations in which one could question the individual. Using the notations introduced above, it is easy to establish the following equality:


1 \[
P\left(c(g)\ =\ {{{\rm c}}_{{\rm i}}}\right)\ =\ \underset{j=1}{\overset{n}{\sum}}\,\ P\left(K(g)\ =\ {{{\rm c}}_{{\rm j}}}\right)\ P\left(E(g)={{{\rm c}}_{{\rm i}}}-\,{{{\rm c}}_{{\rm j}}}\ |K(g)\ =\ {{{\rm c}}_{{\rm j}}}\right)
\]

In this equation, c_1_, c_2_ …c_j_ … c_n_ are the n available degrees of confidence. This equality can also be written in vector form. I will omit in the following the reference to g, use bold letters for vectors and matrices, the sign ‘ will designate the transpose, and I will note


\[
\begin{array}{l}
\boldsymbol{c}\ \, = (P(c = {c_1}),\,\,P(c = {c_2}) \ldots P(c = {c_n}))\\
\boldsymbol{X} = (P(R = 1,\,\,c = {c_1}),P(R = 1,\,\,c = {c_2}) \ldots P(R = 1,\,\,c = {c_n}))\\
\boldsymbol{E} = \left[ {\begin{array}{*{20}{c}}
{P(E = {c_1} - {c_1}|K = {c_1})}& \cdots &{P(E = {c_1} - {c_n}|K = {c_n})}\\
\vdots &{\ldots}& \vdots \\
{P(E = {c_n} - {c_1}|K = {c_1})}& \cdots &{P(E = {c_n} - {c_n}|K = {c_n})}
\end{array}} \right]\\
\boldsymbol{K} = (P(K = {c_1}),\,P(K = {c_2}) \ldots P(K = {c_n}))
\end{array}
\]

The equality (1) can be rewritten in the following way


\[
\boldsymbol{c^{\prime} = E\ K^{\prime}}
\]

In this equality, only **c** is known or at least can be estimated from the proportions of answers given with the corresponding confidence degree. We can, however, establish a second useful equality, via a weak hypothesis on the statistical dependencies between *R(g)* and *E(g)*.

Since the answer *c = c_i_* corresponds to the n exclusive situations,

{*K = c_1_* and *E = c_i_ – c_1_*} or {*K = c_2_* and *E = c_i_ – c_2_*} … or {*K = c_n_* and *E = c_i_ – c_n_*}, we can write


\[
P\left(R\ =\ 1,\ c\ =\ {{c}_{i}}\right)\ =\ {\sum}_{j=1}^{n}P\left(R=1,\ K={{c}_{j}},\ E\ =\ {{c}_{i}}-{{c}_{j}}\right)
\]

that is to say


\[
\begin{array}{l}
P(R\ =\ 1,\,\,c\ =\ {c_i})\ =\ \sum\nolimits_{j\ =\ 1}^n {P(R\ =\ 1|K\ =\ {c_j},\,\,E\ =\ {c_i} - {c_j})} \\
P(K\ =\ {c_j},\,\,E\ =\ {c_i} - {c_j})\\
P(R\ =\ 1,\,\,c\ =\ {c_i})\ =\ \sum\nolimits_{j\ =\ 1}^n {P(R\ =\ 1|K\ =\ {c_j},\,\,E\ =\ {c_i} - {c_j})} \\
P(E\ =\ {c_i} - {c_j}|K\ =\ {c_j})P(K = {c_j})
\end{array}
\]

And under the assumption


\[
P\left(R=1|K\ =\ {{c}_{j}},\ E={{c}_{i}}-{{c}_{j}}\right)\ =\ P\left(R=1|K={{c}_{j}}\right)
\]

the equality becomes


2 \[
\begin{array}{*{35}{l}}
P\left(R\ =\ 1,\ c\ =\ {{c}_{i}}\right)\ =\ {\sum}_{j=1}^{n}P\left(R\ =\ 1|K\ =\ {{c}_{j}}\right) \\
P\left(E\ =\ {{c}_{i}}\ -\ {{c}_{j}}|K\ =\ {{c}_{j}}\right)\ P\left(K\ =\ {{c}_{j}}\right) \\\end{array}
\]

What does the assumption mean? Simply that the probability of giving a good answer depends on the LOK, that is, the state of knowledge in which one finds oneself, but not on the way one estimates that state of knowledge (see discussion below). Under this hypothesis, the previous equality can be expressed, in vector form, as follows:


\[
\boldsymbol{X^{\prime} = E{{D}_{x}}\ K^{\prime}}
\]

with ***D_x_*** = diag (*P*(*R* = 1|*K* = *c_1_*), *P*(*R* = 1|*K* = *c_2_*) … *P*(*R* = 1|*K* = *c_n_*)

In this new equality, the vector ***X*** is known or at least can be estimated as can the diagonal matrix ***D_x_***. It is tempting to write *P*(*R* = 1|*K* = *c_i_*) = *c_i_* because, by definition, in a state of knowledge c_i_, the subject succeeds with a probability c_i_. However this shortcut is unsatisfactory with multiple-choice questions. For example, it is logical to assume that when a subject has no knowledge at all of the topic covered by the question, they still give a correct answer with a probability 1/m where there are m alternatives. In the 2AFC situation I will use to illustrate the model, the c_i_ values are in the set {0.5, 0.6, 0.7, 0.8, 0.9, 1}. A correction for guessing is not necessary with these values as the probability of getting a good answer by guessing is incorporated into the confidence.

Note that the true score model could also be presented as a mixture model. In this formulation, an observation is defined by *c* and *R*: a level of confidence attached to a response that is true or false (*c = c_j_, R* = 1 or 0). This means the observation comes from a multinomial distribution with n (the number of confidence levels) * 2 (response true or false) categories. However, the observation can also be seen as sampled from one of M mixture components corresponding to the different knowledge states and responses. In our setting, M = n * 2 since there are also n possible knowledge states and two possible responses (true or false). Simple algebra makes it possible to establish that, under our conditional independence hypothesis, the distribution in each subpopulation is again multinomial with parameters defined by the different *P(E = c_i_ – c_j_)* (for i = 1, …n) in subpopulations defined by (*K = c_j_, R = 1*) and (*K = c_j_, R = 0*)). The corresponding mixture weights are given by *c_i_ P(K = c_j_)* for the subpopulation defined by (*K = c_j_, R = 1*) and *(1-c_i_) P(K = c_j_)* for the subpopulation defined by (*K = c_j_, R = 0*).

### Maximum likelihood estimates of the different parameters

Different types of biases in confidence assessment perturb the estimation of the LOK, resulting in a mixture of overestimations at some LOK and of underestimations at others. An observed confidence of 70% can be an underestimation of an LOK of 80% or an overestimation of an LOK of 60%. The two basic formulas established above, taken together as a system of equations, make it possible to disentangle these effects. Unfortunately, the number of unknown parameters – the matrix **E** and the LOK vector **K** – is larger (when n > 2) than the number of system equations. More precisely, we have 2n – 1 independent equations (as the sum of the *P(c = c_i_)* is 1, one equation is redundant), and n (n–1) parameters to be estimated in **E** and n–1 in **K**, again taking into accounts relations between probabilities whose sum must be equal to 1. In total, there are 2n–1 relations for (n–1) (n+1) parameters. Therefore, there is indeterminacy when n > 2. To proceed further, some constraints on the form of **E** must be added. Different possible forms are given in the next paragraph. Modeling **E** to reduce its estimation to the estimation of a limited number of parameters makes the problem solvable.

If I note α_1_, α_2_, …, α_q_ the parameters needed to define **E**, with q < n so that the number of equations exceeds the number of parameters, we are faced with the following system of nonlinear equations (the last equation of (3) being redundant):


3 \[
{\boldsymbol X}^{\prime}\ =\ {\boldsymbol E}\ \left({\alpha}_{1},{\alpha}_{2},\ldots {\alpha}_{q}\right)\ {\boldsymbol D}_{\boldsymbol x}\ {\boldsymbol K}^{\prime}
\]

4 \[
{\boldsymbol c}^{\prime}\ =\ {\boldsymbol E}\ \left({\alpha}_{1},\ {\alpha}_{2},\ldots {\alpha}_{q}\right) {\boldsymbol K}^{\prime}
\]

Note that from **X** and **c**, we can easily derive the joint distribution of accuracy and confidence (R and c). Indeed *P(R = 1, c = c_i_) =* ***X****_i_* and *P(R = 0, c = c_i_) =* ***c****_i_ –* ***X****_i_* . Given the data collected on N items for a respondent, we can therefore derive the maximum likelihood values of the parameters of the model. Given that the joint distribution of *R* and *c* is a multinomial distribution, this requires to maximize the probability of the sample under the null hypothesis (here defined by the model). I will note N_1i_ the observed number of correct responses with confidence c_i_ in the sample and N_0i_ the observed number of wrong responses with confidence c_i_. The likelihood function to maximize is thus given by


5 \[
N!\mathop \prod \limits_1^n \frac{{{\boldsymbol{X_i}}}}{{{N_{1i}}}}\frac{{({\boldsymbol{c_i}} - {\boldsymbol{X_i}})}}{{{N_{0i}}}}
\]

A likelihood ratio test can be carried out with the statistics


6 \[
- 2\left(\mathop \sum \limits_1^n {N_{1i}}\ln \left(N\frac{{{X_{1i}}}}{{{N_{1i}}}}\right) + \mathop \sum \limits_1^n {N_{0i}}\ln \left(N\frac{{({{\boldsymbol c}_i} - {{\boldsymbol X}_{1i}}}}{{{N_{0i}}}}\right)\right)
\]

The distribution of this statistics, as N increases, converges to a chi-square distribution with (n – q) degrees of freedom. Maximizing the likelihood function involves optimizing a nonlinear function. This can be achieved, for instance, using the “fmincon” function in MATLAB. Since the parameters to be estimated are probabilities, constraints must be introduced in the optimization process.

### Assumptions on the matrix E

If we assume that the matrix E is the identity matrix, then *P*(*E* = 0| *K* = *c_i_*) = 1 for all i. This implies that there is no error E regardless of the level of knowledge. Consequently, confidence reports can be taken at face value. To benefit from the proposed approach, some errors in the confidence ratings should be allowed. This leads to the consideration of four possible models in the paper. Other constraints could be imposed on the errors, depending on the biases of interest. The models have been specified to capture some empirical regularities (e.g., overconfidence). I will assume here that we have 6 possible confidence levels: 50% 60% 70% 80% 90% 100% (n = 6).

#### Model S

The first model is the simplest one. It postulates that

1. an LOK level is only confused with an adjacent one (the errors of estimation on confidence are small);
2. regardless of the LOK, the probability of estimating it correctly is always equal to α;
3. the errors committed when K(g) is 60% or 70% are not different from those committed when K(g) is 80% or 90% (no hard-easy effect);
4. the probability of underestimating by 10% (β when K(g) *is* lower than 100%) is different from the probability of overestimating by 10% (1–α–β when K(g) is greater than 50%).

Let us remark that the first assumption is demanding and will prove to be compatible with only part of the students. Those assumptions lead to the following error matrix.


\[\boldsymbol{E}\ =\ \left(\begin{array}{c} \alpha & \beta & 0 & 0 & 0 & 0 \\
1-\alpha & \alpha & \beta & 0 & 0 & 0 \\
0 & 1-\alpha -\beta & \alpha & \beta & 0 & 0 \\
0 & 0 & 1-\alpha -\beta & \alpha & \beta & 0 \\
0 & 0 & 0 & 1-\alpha -\beta & \alpha & 1-\alpha \\
0 & 0 & 0 & 0 & 1-\alpha -\beta & \alpha \\\end{array}\right)\]

The model will be called model S(imple). The total number of parameters is 7 (5 for K plus 2 for E) and the number of equations is 11. The problem is tractable.

#### Model H

In the second model, the second hypothesis made above has been dropped. The error matrix E has the following form.


\[\mathbf{E}\ =\ \left(\begin{array}{c} \alpha & \beta & 0 & 0 & 0 & 0 \\
1-\alpha & \alpha & \beta & 0 & 0 & 0 \\
0 & 1-\alpha -\beta & \alpha & \gamma & 0 & 0 \\
0 & 0 & 1-\alpha -\beta & \alpha & \gamma & 0 \\
0 & 0 & 0 & 1-\alpha -\gamma & \alpha & 1-\alpha \\
0 & 0 & 0 & 0 & 1-\alpha -\gamma & \alpha \\\end{array}\right)\]

In this model, α is the probability of making no error in the estimation of the LOK, β the probability of underestimating the LOK by 10% when the LOK is low (e.g. if the LOK is 60%, the given confidence will be 50%), and γ the probability to underestimate it by 10% when the LOK is high but lower than 100% (e.g., if the LOK is 90%, the confidence will be 80%). This matrix is similar to the one used in model S but the errors committed when K(g) is 60% or 70% may be different from those committed when K(g) is 80% or 90%. This allows for the identification of a hard/easy effect and the model is consequently called model H(ard/easy).

#### Model O

The model O(bstination) is also a variant of the model S. Unlike model S, I no longer assume that “regardless of the LOK, the probability of estimating it correctly is always equal to α″. This is clearly a strong simplification. Indeed, for a person who is either totally ignorant (LOK = 50%) or certain of the response (LOK = 100%), the probability of correctly estimating the LOK (noted γ) should differ from the probability α of correctly estimating the other LOK values. This leads to model O with the following error matrix.


\[\mathbf{E}\ =\ \left(\begin{array}{c} \gamma & \beta & 0 & 0 & 0 & 0 \\
1-\gamma & \alpha & \beta & 0 & 0 & 0 \\
0 & 1-\alpha -\beta & \alpha & \beta & 0 & 0 \\
0 & 0 & 1-\alpha -\beta & \alpha & \beta & 0 \\
0 & 0 & 0 & 1-\alpha -\beta & \alpha & 1-\gamma \\
0 & 0 & 0 & 0 & 1-\alpha -\beta & \gamma \\\end{array}\right)\]

#### Model A

The model A(ssertiveness) is strongly inspired by the replication context used to test the models. It allows for the observation of how reported judgments are more or less extreme on the confidence scale, a kind of Extreme Response Style (Deng et al., 2018). As previously mentioned, when a test is repeated, other kinds of errors, not linked a priori to calibration, can emerge. Model O can already be viewed from that perspective. When students are invited to reassess their confidence in a response after some time, their behaviour may differ when the initial confidence was 50% or 100% (often corresponding to extreme values of states of knowledge) compared to when the confidence is between these two extremes. This behavior is easily detectable with repeated measures. In model A, a similar focus is placed on the repetition error. Some students may be “attracted” to the value 100%, exhibiting a kind of assertiveness. In this model, the first assumption made for models S, H and O – that an LOK level is only confused with an adjacent one (the errors of estimation on confidence are small) – is modified to allow “jumps” from any level of LOK to a confidence of 100%. For example, if I have 70% confidence in my response, in the first three models, this should correspond to an LOK of 60%, 70% or 80%. In none of those models could I jump, in a new trial, to a confidence of 100%. This is now possible with model A. Its error matrix is given below.


\[\mathbf{E}\ =\ \left(\begin{array}{c} \alpha & \beta & 0 & 0 & 0 & 0 \\
1-\alpha -\delta & \alpha & \beta & 0 & 0 & 0 \\
0 & 1-\alpha -\beta -\delta & \alpha & \beta & 0 & 0 \\
0 & 0 & 1-\alpha -\beta {}^\circ -\delta & \alpha & \beta & 0 \\
0 & 0 & 0 & 1-\alpha -\beta -\delta & \alpha & 1-\alpha \\
\delta & \delta & \delta & \delta & 1-\alpha -\beta & \alpha \\\end{array}\right)\]

### Example

Before developing an empirical example from a test carried out in 2021, analyzing the responses of a single individual can already illustrate the potential of the approach. The task was a 2AFC test consisting of 60 questions, with 6 possible degrees of confidence: 50%, 60%, 70%, 80%, 90%, 100% (n = 6). I selected a student whose response profile is not overly simplistic. The data are provided in Table 1.

**Table 1:** Number of Correct and Incorrect Responses by Level of Confidence for a Respondent on a 60 2AFC Questionnaire.


	50%	60%	70%	80%	90%	100%

Correct	4	2	4	4	10	23

Incorrect	2	2	3	1	3	2


**Figure 2:**
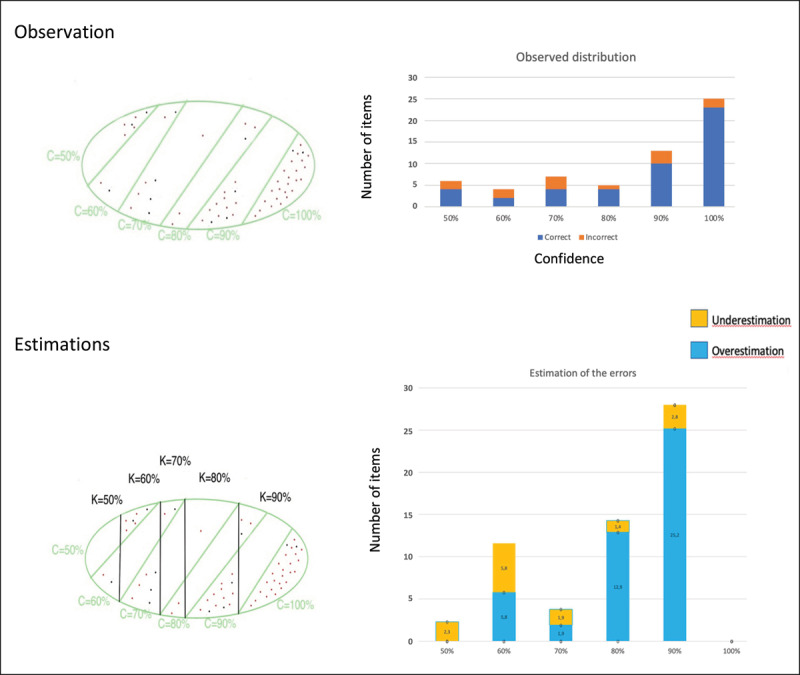
Two Different Representations of Observed and Estimated Data. *Note*. The number of correct/incorrect responses as a function of confidence represented in 2 different ways. Bottom panels: the distribution of the LOK, superposed with the confidences given by the respondent on the left, and with the highlighting of under and overestimations on the right.

Given the distribution of the responses (see below how the different models were adjusted to the data), model H is applied to these data (chi2 = 0.63 with df = 3) and the results are presented in Figure 2. The two top panels represent the observed data in two different ways: a Venn diagram (on the left) and a more traditional bar chart (on the right) showing the number of correct and incorrect answers for each confidence level. The distribution of the LOKs and their overlap with the given confidences are represented in the bottom left panel. The bottom right panel shows the proportion of over and underestimations for each level of knowledge.

Note that once the errors have been taken into consideration, the knowledge profile of the respondent has changed. According to model H, the respondent consistently underestimates or overestimates their level of knowledge. The model also predicts that if the test is replicated 74% of the confidence levels (for identical answers) provided by the subject should remain identical assuming independent errors for the same levels of knowledge (LOKs). Please note that this prediction does not account for the possibility of altered responses in the second trial. The observed percentage of identical confidence levels (calculated from the 43 items with unchanged responses) is 84%. Thus, the model provides an insight into the expected outcomes in a replication. Further discussion on this intriguing property of the approach will follow.

## Materials & Methods

### Data and objectives of the study

Data collected during an experiment conducted by Leclercq in 2021 (Leclercq, 2025) have been analyzed. Forty-one master’s students (80% women) were presented with 60 2AFC general-knowledge items: 30 questions on animal cries and 30 questions about capital cities. For instance, students were asked to indicate whether the statement “The duck cackles” was true or false and to associate their response with a degree of confidence chosen from six possible values: 50%, 60%, 70%, 80%, 90%, and 100%. The test was repeated two hours later. The data were anonymized, and students were informed at the time that the data could be used for research purposes. They had the option not to take the test. This experiment was designed with two main objectives: to keep the students in the same mindset and to minimize the possibility of memorizing the degrees of confidence given in the first trial. For information, Koriat (2013) conducted a similar experiment with 41 students and 60 2AFC questions, but the replication occurred one week later, and he did not attempt to model the errors. This experiment illustrated his theory of self-consistency. The questions were about preferences rather than true/false alternatives (Koriat, 2013).

The replication of the test allowed for the identification of additional features in the students’ judgments.

The objectives of the analysis are twofold:

1. To illustrate the potential of the approach. The students exhibit various misclassification patterns that can be estimated using the models.
2. To demonstrate that analyzing the data from the first trial can identify traits that are only directly observable through replication. Calibration errors are not independent of repetition errors.

### Method

To estimate the miscalibration patterns of each student, I used the four different true score models (S, H, O and A) presented above, each defined by different choices of the error matrix E. The models were adjusted based on the data from the first trial only. Model S is the simplest model, designed to detect possible underconfidence or overconfidence. As previously explained, Model H is a straightforward variation of Model S, necessary to detect a possible hard/easy effect. Model O captures a trend—one not studied in the existing literature, as far as I know—where individuals behave differently under conditions of complete knowledge or total ignorance. As previously explained, Model A is used to capture an attractive effect of the 100% confidence level.

The estimation of a true score model requires, as previously mentioned, the resolution of a system of nonlinear equations under constraints—a challenging task with limited data. The estimates can be unstable, as they depend on the initial values given to the parameters in the iterative process used to solve the equations. Consequently, the risk of erroneously accepting a model based on the chi-square value is high.

To address these difficulties, the following steps were taken:

1. The number of parameters in the models was limited to a maximum of three to define the error matrix E.
2. Fifty runs were performed with the same data but different initial values. The initial values were random, and one of them, when relevant, was the value obtained for a simpler model.
3. The four different models were tested on all the data, and various measures of fit were considered: the traditional likelihood ratio test and the Akaike information criterion (AIC).

For each model, two measures of fit were considered: the statistics for the likelihood ratio test, which is asymptotically distributed as a chi-square, and the AIC, which measures the relative amount of information loss. The AIC is particularly useful for comparing models that are not nested, such as models H, O, and A. These measures of fit were calculated for each student. When relevant, the error patterns detected during the first trial were compared with those observed during the second trial.

## Results

The organization of the Results section is as follows. The first section will present general characteristics of the entire dataset, specifically the responses given by the 41 students during the first trial. The second section will focus on the links between the two replications. The third section will show the results of fitting the four models to the data. The final section will discuss what the models reveal about the second trial.

### The first trial

**Figure 3:**
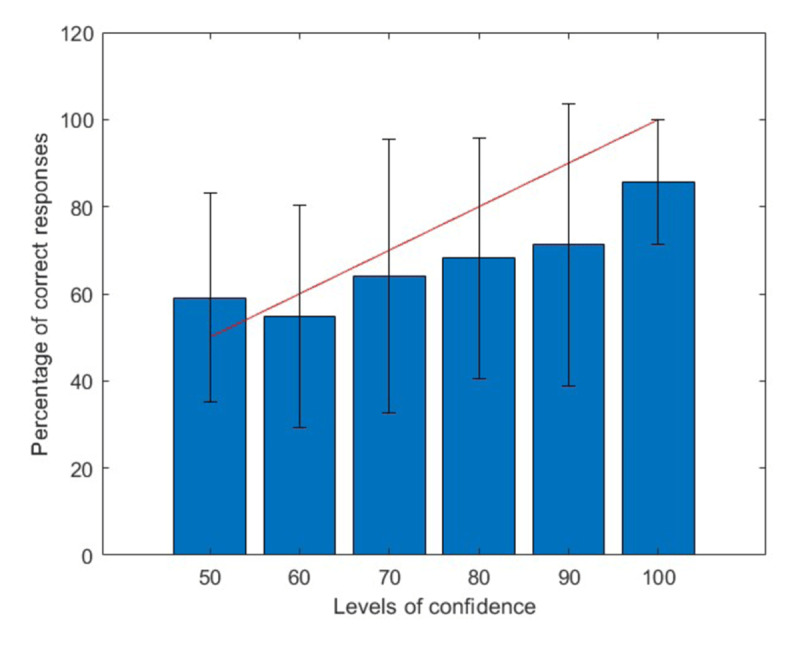
Percentages of Correct Responses as a Function of the Degree of Confidence with Standard Deviations. *Note*. The red line shows what the percentage should be if the students were perfectly realistic.

The mean score, measured as the percentage of correct responses, is 72%, while the average confidence is 78%. Figure 3 illustrates the overall ability to distinguish between different levels of confidence. Except for the 50% confidence level, the proportion of correct responses increases with the level of confidence. The Spearman correlation coefficient between the given confidence levels and the corresponding proportion of correct responses is significantly greater than 0 for 73% of the students at a significance level of 0.05, using the Bonferroni correction. The red line indicates the expected percentage if the students were perfectly realistic. It shows that, on average, the given confidence overstates the actual performance, except at the 50% confidence level. The error bars represent the standard deviations of the percentage of correct responses for each level of confidence, calculated among the students.

Figure 3, as illustrated by the error bars, conceals individual differences. The computation of the indicator of realism (see the section on the measurement of subjective confidence) calculated for each student, makes the heterogeneity of the data more explicit. Realism measures the ability to use confidence appropriately. It is calculated for each student as the mean absolute difference between the used confidence degree and the corresponding proportion of correct responses. It ranges from 0, when the student is perfectly realistic (on average, the proportion of correct responses with confidence x is x/100), to 100, when the student always responds with confidence 100 and is always wrong.

**Figure 4:**
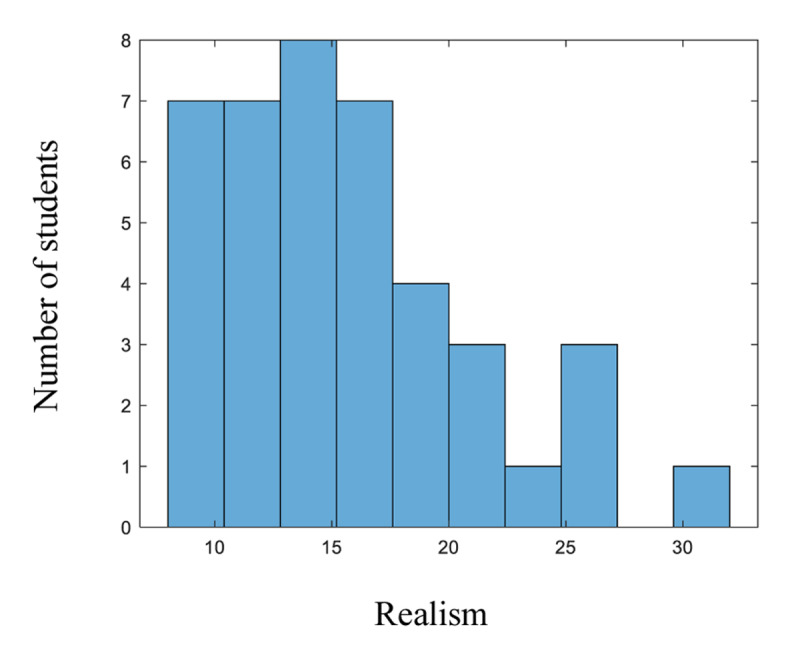
Distribution of the Realism of the 41 Students.

The mean realism is 16, but there are significant variations among students (ranging from 8 to 32). The histogram in Figure 4 shows the distribution of the scores among the 41 students.

The discrepancies between students already suggest that different models will likely need to be adjusted.

### The second trial

On average, students change 20% of their responses (switching from true to false or vice versa). All students change at least one of their 60 responses. In 44% of the cases, students do not change either their response or their confidence. Focusing only on cases where the response does not change, the percentage of changed confidence is 45%. This likely underestimates the number of changes, as some individuals, despite the experiment’s design, may have memorized their confidence levels from the first trial and tried to remain consistent.

**Figure 5:**
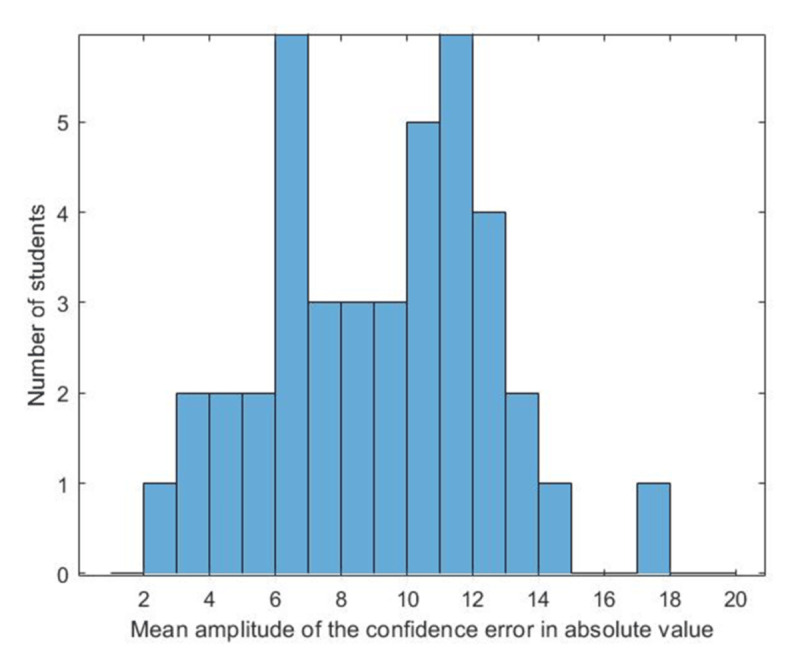
Distribution of the Mean Amplitude of the Confidence Error in Absolute Value among the 41 Students.

When a student changes their response, the average confidence given in the first trial is 68%. Comparing the confidences for unchanged responses between the two trials, we find an average error of –1.45%. This average error, measured in absolute terms, is 9.1%. This indicates that, on average, a student shifts to a contiguous level of confidence during the second test. However, the mean error varies among students. The histogram in Figure 5 shows the distribution of mean confidence errors (in absolute values) among the 41 students.

The Pearson correlation between the confidences given at the first and the second trial (when responses are unchanged) is 0.66.

**Figure 6:**
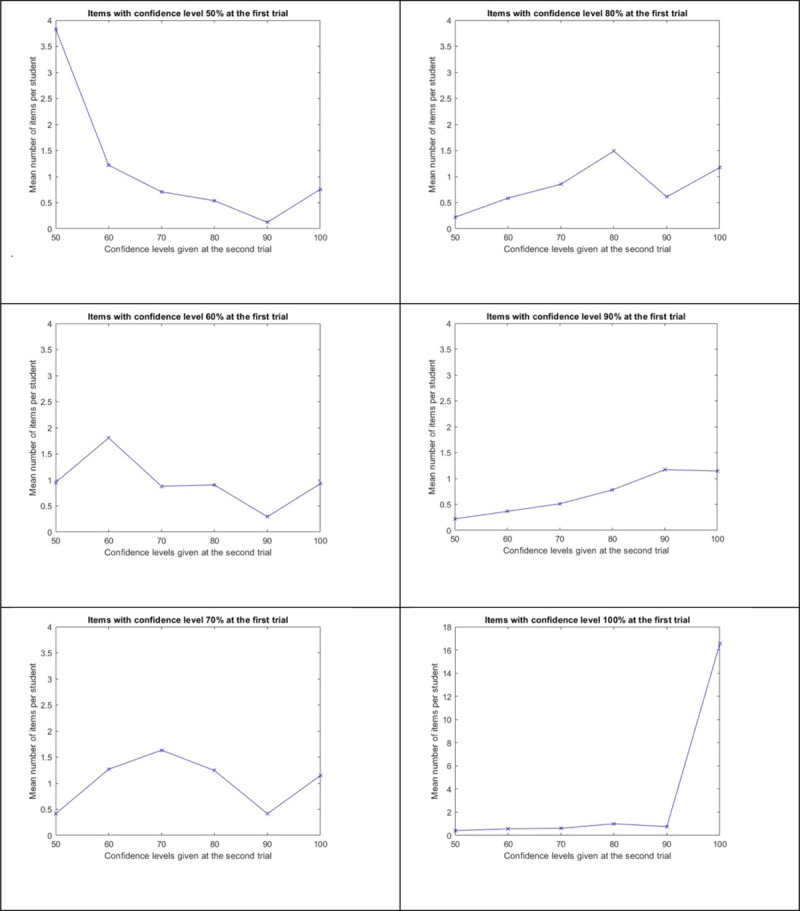
Distribution (in Mean Number of Items per Student) of Confidence at the Second Trial for the Items with Confidence Degrees 50%, 60%, 70%, 80%, 90% and 100% at the First Trial.

A more meaningful way to compare the confidences given at the two trials is to consider bivariate distributions. This approach examines the distribution of the confidences given at the second trial for items where the confidence was fixed—let us say x%—at the first test (with the same response). One expects a distribution centered on x and vanishing on both ends. This can be done for x ranging from 50 to 100. In Figure 6, the top left panel, for instance, considers only items with a confidence degree of 50% at the first trial. For each student, the number of those items with a second trial confidence level of 50%, 60%, 70%, 80%, 90% and 100% are counted. An average of all those counts among students for each second trial confidence level is computed. The means represented in the figure summarize highly variable values among students.

**Figure 7:**
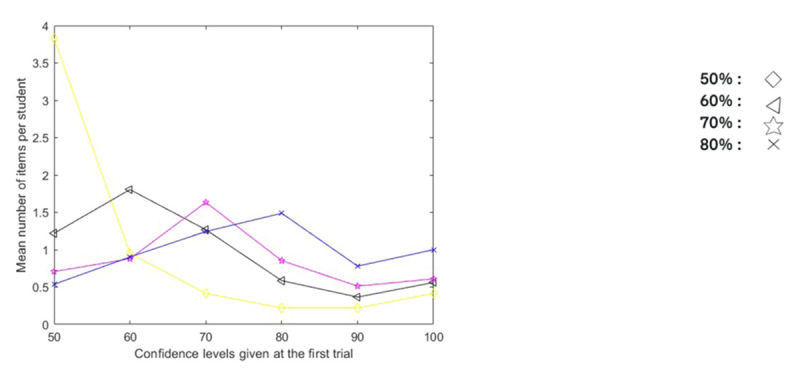
Distribution (in Mean Number of Items per Student) of Confidence Given at the First Trial for the Items with Confidence Degree 50%, 60%, 70%, 80% at the Second Trial.

This analysis reveals several insights:

1. First, the number of items with a confidence level of 100% (one-third of the confidences given at the first trial) is much higher than all the other levels (see the bottom panel on the right). This indicates that students are certain about a substantial number of animal cries and capital cities.
2. As expected, all the distributions are centred (have their mode) around the value given at the first trial.
3. A kind of rebound is observed at the 100% confidence level. This rebound is also observed if we permute the two trials and examine the distribution at the first trial conditional on what has been observed at the second trial. Figure 7 illustrates what happens when the two trials are permuted. This is the “mirror” figure of the ones given above. In this figure, the conditional distributions of confidences at the first trial for the items with confidence degree 50%, 60%, 70%, 80% at the second trial have been superposed. This effect cannot, therefore, be fully attributed to a learning process. Rather, it corresponds to the pattern expected if Model A, as described in the Method section, holds true. A more detailed analysis of this effect is provided in the section titled “Comparison of the Predictions of the Models with Observations from the Second Trial.”

Figures 6 and 7 clearly show that the confidences given at the second trial are not identical to those given at the first trial.

### Fitting of the 4 models

For model S, the mean chi-square value, calculated among the 41 students with 4 degrees of freedom (df), is 17.20. It fits the data of 13 students at the 0.05 significance level (and 20 students at the 0.01 significance level). I will refer to these 13 students as S-students. As expected, this simple model is only compatible with a subset of students.

The slightly more complex H model naturally has a better fit. The mean chi-square value, with 3 df, is 8.30. Twenty-two students respond in a way that is not incompatible with the model at the 0.05 significance level (and 33 students at the 0.01 significance level). This indicates that allowing for a level of error that depends on the LOK (rather than a general tendency to overestimate or underestimate, as in model S) improves the fit. In fact, out of the 22 H-students, 19 have a value of β > γ. This means that the majority of H-students exhibit the opposite of a hard/easy effect, showing a higher tendency to underestimate themselves when they do not know.

Model O, of which Model S constitutes a special case, yields a mean chi-square value of 8.19 with 3 degrees of freedom. It fits the data of 21 students at the 0.05 significance level (and 34 students at the 0.01 significance level). The main interest of this model, as already mentioned, is that its predictions can easily be compared with what is observed during the second trial.

Model A, like models H and O, is a refinement of model S. The mean chi-square value, with 3 df, is 7.37. It fits the data of 21 students at the 0.05 significance level and 32 students at the 0.01 significance level. The predictions made by this model can be validated using the data from the second trial. This process is detailed in the following section.

For 3 out of the 41 students, none of the models fit as measured by the chi-square test, even at the 0.01 significance level. Their behavior cannot be described by our four error matrices. Their use of confidences appears to be “chaotic,” as there is no relation between the accuracy of their responses and the associated confidences. For each student, a Pearson correlation coefficient between the percentage of correct responses for each confidence level and that level—expected to be 1 if there were no error—can be calculated. The values for two of these three students are –0.56 and 0.01, respectively. The third student uses only two confidence levels (for more than 90% of the responses).

Table 2 provides a comparison of the four models using the Akaike Information Criterion (AIC) for the remaining 38 students. For each student, the model that minimizes the information loss according to the AIC is selected.

**Table 2:** Identification of the Best Model with the AIC.


	MODEL S	MODEL H	MODEL O	MODEL A

Number of students	16	12	4	9


### Comparison of the previsions of the models with what is observed at the second trial

Models O and A, estimated using only the data from the first trial, exhibit characteristics of the students that should be apparent in the replicated data. Model O assumes that the probability of making no error when K = 100% and K = 50% is different from the probability of making no error for other levels of knowledge. However, the probability of making no error when the LOK is 50% or 100% (measured by γ in model O) can be approximated using the data collected during the second trial. Specifically, one can count the number of times the confidence values of 50% and 100% are repeated in the second trial (with the same answer). This estimate, constructed from these counts, is not exactly what we want because confidences are not LOKs, and consequently, a confidence of 100% given in the second trial can come from an LOK of 90%, for instance. Nevertheless, this should provide a good approximation.

I calculated the proportion of times each confidence level is repeated in the second trial. I averaged the confidences of 50% and 100% (noted) *γ∼* and the confidences of 60%, 70%, 80%, and 90% (noted as *∼α*). These two statistics are rough approximations of the parameters γ and α of model O. One could expect that for O-students who are not S-students (9 students), the differences are larger in absolute terms than for S-students. This is indeed what is observed. The mean difference in absolute values is 0.61 (std = 0.16) for those who fit model O without fitting model S and 0.21 (std = 0.15) for S-students.

**Figure 8:**
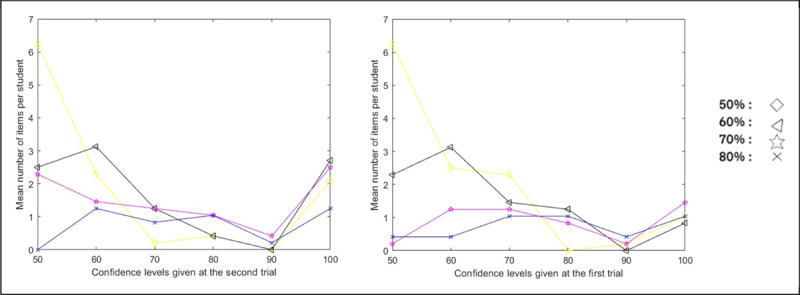
Conditional Distribution of Confidences at One Trial Given Confidence Levels of 50%, 60%, 70%, and 80% at the Other Trial. *Note*. Left panel: distribution (in mean number of items per student) of confidence at the second trial for the items with confidence degrees 50%, 60%, 70%, 80% at the first trial, for students significantly attracted by the confidence level 100% according to model A. Right panel: the same distributions when the two trials are permuted.

A similar analysis can be carried out for model A. Do we observe an attraction to 100% confidence (in the second trial) – the so-called rebound effect – for the 8 A-students whose *δ* values are significantly different from 0 (i.e. A-students that are not S-students)? An initial examination of the conditional distribution of confidences given at the second trial for items with confidence levels of 50%, 60%, 70%, and 80% at the first trial clearly shows that this subset of 8 students exhibits a strong rebound effect. This is illustrated in Figure 8 in two different ways : in the left panel, with the conditional distributions of confidence levels given at the second trial and in the right panel, with the same distributions when the two trials are permuted.

As I did for the “Obstination effect”, I can construct, with the replicated data, a rough approximation of the rebound as measured by the parameter *δ* of the model A. The probability (measured by *δ* in model A) to jump to a 100% confidence level when the LOK is 50%, 60%, 70% or 80% can indeed be approximated with the data collected during the second trial. One can count for those 4 levels of confidence the proportion of times they are changed to 100% at the second trial. The mean value of those 4 proportions could be considered as a rough estimation of *δ*. Again, this estimate is not exactly what we want because confidences are not LOK and consequently a confidence of 80% given at the first trial can come from an LOK of 70 or 90% for instance. But this should nevertheless provide a good approximation. The Pearson correlation between this estimate and the *δ* calculated on the first trial with model A is 0.77. This result shows once more the capacity of the approach to predict what will happen when the data are replicated.

## Discussion

The present study had three primary objectives: (1) to establish a true score model for the estimation of the LOK, (2) to identify potential links between miscalibration errors and errors observed in replicated data, and (3) to demonstrate the approach and the links through an application to a dataset of replicated data from 41 subjects. Each of these objectives will be discussed in turn.

### True score model

The assumption of a true score to be estimated—a concept widely utilized in the literature—is foundational. True score models have been employed for decades in psychometrics (Lord & Novick, 1968), yet the application of this concept to LOK has not been previously attempted, to the best of my knowledge. One might argue that LOK is either an out-of-focus reality or a mere construct, rendering the proposed model irrelevant: individuals do not have direct access to the correctness of their responses. Instead, they rely on heuristics tailored to their environment to assess their confidence. My hypothesis diverges slightly from this perspective. I propose that individuals’ confidence should not be taken at face value but rather as indicative evidence of their true LOK.

In a traditional true score model, the error is typically considered independent of the true ability, in this case, the LOK. However, I did not make this assumption and instead proposed a lighter hypothesis of conditional independence. How does this align with existing explanatory models? Let us recall the assumption: given a question belonging to a specific LOK, the correctness of the response is independent of the error made in estimating that level. In other words, the errors are, on average, the same for both correct and incorrect answers for a given LOK. The error in estimating the LOK depends on the proposed response only through the LOK. For approaches based on a cue elicitation process, which encompasses most existing models, this implies that the cue retrieval activity within a LOK does not depend on the correctness of the response. The reference framework emerging in the thinking process, including the number of cues, beliefs, and their validity, defines the LOK but does not vary according to the accuracy of the choice made. This seems a reasonable assumption.

The models also assume that individuals experience a degree of confidence in the truth of what they know. However, the number of correctness feelings they can have corresponds to the number of LOK states requested by the experimenter. This cannot always be true. It is assumed that the experimental context frames the correctness feelings of individuals. This only makes sense under certain conditions, as studied by Leclercq (2016). For instance, he demonstrated that the way confidence degrees are formulated (with words or different percentage scales) impacts the accuracy of the responses. Koriat (1993) also postulated that “the feeling of knowing is parasitic on the processes involved in attempting to retrieve the target, relying on the accessibility of pertinent information.”

### Links between miscalibration patterns and replication errors

Two types of errors have been considered in this paper. The first type, calibration error, measures the discrepancy between the confidence expressed by the respondent and what I have termed the LOK. This discrepancy has been extensively explored in numerous studies, as discussed in the introduction, and is the central issue in calibration research. The second type of error, replication error, has received less attention in the literature.

Replication of a test, under conditions that allow for the comparison of given confidences to the same questions and responses, reveals another type of discrepancy between repeated observations. The true score approach predicts that replication will only affect the calibration error, not the true score, i.e., the LOK. In other words, for a given item and respondent, the only source of variation is the calibration error. Thus, replication and calibration errors are identical. This strong, postulated link between the two types of errors explains the approach’s ability to predict, from a single test, what will occur in a replicated test. This also enables the detection of specific miscalibration patterns (such as obstinacy and assertiveness traits) that are only identifiable through repeated measurements.

The use of repeated measurements should allow for testing the existence of a possible consistency effect (see the section on biases in confidence assessments). However, this requires more than one replication of the test and cannot be accomplished with the data used in this study. A more formal approach to the effect can be proposed based on the modeling of errors, but this is beyond the scope of the current paper.

### Application of the model

The analysis of the data reaffirms the general ability to monitor the accuracy of answers in a general knowledge task (see Figure 3). However, this ability is not uniformly distributed across the population, and various types of biases exist.

A simple model, assuming errors of limited amplitudes (10%), has shown a fairly good fit with the data. Excluding three students whose data were difficult to model, Model H fits the data of approximately 87% of the sample at the 0.01 level. The analysis of the dataset has thus confirmed the existence of calibration errors and a predominance of overestimation, which are well-known phenomena in tasks assessing general knowledge.

The interaction effect highlighted by Model H is not a hard/easy effect but rather a “polarization” effect of the confidences. Nearly half of the students exhibit a higher tendency to underestimate themselves when they are uncertain.

Three out of the four models under consideration—namely models S, H, and O—assume that calibration errors do not exceed a maximum amplitude of 10%. Nonetheless, this assumption is not consistently satisfied by all students, even among those who demonstrated a good fit with these models (referred to as SHO students). For these students, if the assumption holds, the discrepancy between confidence ratings provided during the first and second trials should remain below 20%. This can be validated by comparing confidence ratings given in instances where the responses remain identical. Among the 1212 paired ratings submitted by the 25 SHO students, 13% exceeded this threshold. Several factors might account for this; for instance, students may prioritize their responses over the associated confidence ratings. Predictably, this proportion rises to 19% among the A-students who are not categorized as SHO students (88 paired responses provided by 2 students). Indeed, for these students, model A accommodates higher calibration errors.

The application of the true score approach enabled the identification of various miscalibration patterns within a small sample of students. This underscores the necessity of exercising caution with traditional “population” approaches that focus on general patterns such as overestimation or the hard/easy effect. Metacognitive skills are diverse, and those interested in metacognitive regulation often need to compare metacognitive biases before and after specific training (see, for instance, Mattes & Pieschel, 2022). A detailed understanding of these biases will likely enhance the comprehension of training effects.

However, the design and adjustment of the models are not straightforward. First, regarding the design, constraints are imposed on the error matrix E. Since the elements of E are probabilities, the columns must sum to 1. This necessitated distinguishing between events where K = 50% or 100% and other events. In Model S, for instance, β represents the probability of underestimating in all situations except when K = 100%. Second, with a limited number of items and numerous parameters, estimation errors should not be underestimated. This can lead to difficulties in choosing among alternative models. Additionally, the iterative method used to maximize the likelihood functions requires initial values, and the results can be sensitive to these initial values (due to the existence of local maxima). This necessitates multiple runs with different initial values, a well-known practice for algorithms requiring iteration, such as neural network adjustments.

## Conclusions

This study has demonstrated that a simple true score model applied to confidence degrees is tractable under a light assumption of conditional independence (the error in the estimation of confidence degrees depends on the proposed response only through the LOK) and simple constraints imposed on the way confidence errors are distributed. The model allows for the correction of given confidences by accounting for miscalibration effects. Maximum likelihood estimates of these effects can be calculated, which requires solving a system of nonlinear equations under constraints for each subject.

The model does not assume a specific cognitive process and therefore cannot specify how judgments are biased. If the underlying process involves the extraction of cues from a pool of representations, the latent trait LOK can be considered as a proportion of representations favoring a response, with errors linked to the sampling of information from the pool in a given context.

The study of repeated measurements of confidence enabled testing the model and demonstrating how repetition errors are linked to calibration errors. A detailed analysis of miscalibration patterns allowed for the prediction of effects observable only through test replication. This should broaden the scope of already identified biases, extending beyond traditional over/underestimation and hard/easy effects.

Models should be handled with great care. The estimation of certain parameters could be based on very few observations when tests have a limited number of questions. Therefore, errors should be assessed with a large number of questions whenever possible. Additionally, models with few parameters should be favored.
